# *Urtica dioica*: Anticancer Properties and Other Systemic Health Benefits from In Vitro to Clinical Trials

**DOI:** 10.3390/ijms25137501

**Published:** 2024-07-08

**Authors:** Marc Abi Sleiman, Maria Younes, Roy Hajj, Tommy Salameh, Samir Abi Rached, Rimane Abi Younes, Lynn Daoud, Jean Louis Doumiati, Francesca Frem, Ramza Ishak, Christopher Medawar, Hassan Y. Naim, Sandra Rizk

**Affiliations:** 1 Department of Natural Sciences, Lebanese American University, Byblos P.O. Box 36, Lebanon; marc.abisleiman@lau.edu (M.A.S.); maria.younes01@lau.edu (M.Y.); roy.elhajj01@lau.edu (R.H.); tommy.salameh@lau.edu (T.S.); samir.abirached@lau.edu (S.A.R.); rimane.abiyounes@lau.edu (R.A.Y.); lynn.daoud@lau.edu (L.D.); jeanlouis.doumiati@lau.edu (J.L.D.); francesca.frem@lau.edu (F.F.); ramza.ishak@lau.edu (R.I.); christopher.medawar@lau.edu (C.M.); 2 Department of Biochemistry, University of Veterinary Medicine Hannover, 30559 Hannover, Germany

**Keywords:** *Urtica dioica*, natural product, pharmacological properties, anticancer, health benefits

## Abstract

While conventional medicine has advanced in recent years, there are still concerns about its potential adverse reactions. The ethnopharmacological knowledge established over many centuries and the existence of a variety of metabolites have made medicinal plants, such as the stinging nettle plant, an invaluable resource for treating a wide range of health conditions, considering its minimal adverse effects on human health. The aim of this review is to highlight the therapeutic benefits and biological activities of the edible *Urtica dioica* (UD) plant with an emphasis on its selective chemo-preventive properties against various types of cancer, whereby we decipher the mechanism of action of UD on various cancers including prostate, breast, leukemia, and colon in addition to evaluating its antidiabetic, microbial, and inflammatory properties. We further highlight the systemic protective effects of UD on the liver, reproductive, excretory, cardiovascular, nervous, and digestive systems. We present a critical assessment of the results obtained from in vitro and in vivo studies as well as clinical trials to highlight the gaps that require further exploration for future prospective studies.

## 1. Background

Since the 20th century, technological advances in microscopy and chemical engineering, the emergence of highly resourceful pharmaceutical companies, the accessibility of novel sources for medicinal compound extraction, and the considerable increase in research and development spending have expanded the pool of available drugs [1,2]. However, even prior to the discovery of the microscopic world, primitive and ancestral forms of medicines predominantly relied on medicinal plants and their therapeutic properties.

To this day, plants have retained their place in both conventional and alternative medicines given the identification of their phytoconstituents with demonstrable benefits against various diseases [3]. As an alternative option, plants are relatively inexpensive, have remarkable chemical diversity, and circumvent cultural or religious controversies that would otherwise hinder the accessibility of primary care in the developing world [4]. One such plant is *Urtica dioica* (UD), better known as the nettle plant, common nettle, stinger, or stinging nettle. UD is a herbaceous plant of the *Urticaceae* family that belongs to a diverse taxonomic group, the details of which are outlined in Table 1 [5,6]. As for its nomenclature, the Latin name “*Urtica*” translates into “to burn”, whereas its common name, nettle, is derived from the Anglo-Saxon word “noedl” meaning needle. Fittingly, this flowering plant is characterized by stinging hairs, located on both the stems and leaves, that cause a burning sensation when rubbed against the skin. This irritation is majorly due to the release of a fluid containing acetylcholine, formic acid, acetic acid, serotonin, histamine, and leukotrienes among other substances responsible for this effect [2,7,8].

**Figure 1:**
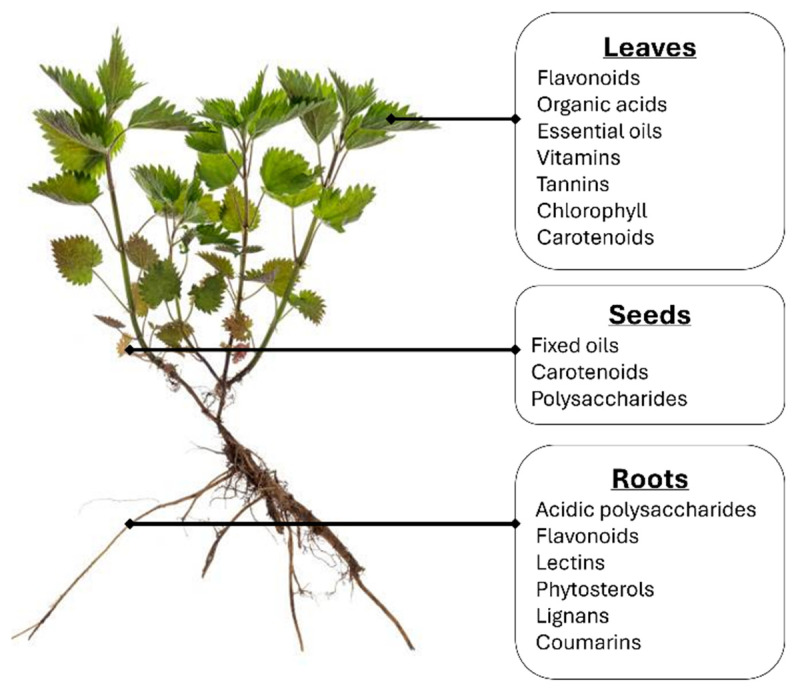
Overview of the chemical composition of the nettle plant.

The use of common nettle in traditional medicine in the form of crude dried powder, tea infusions, decoctions, or fresh juices dates back several centuries [6,9]. However, modern interest has shifted to the use of UD extracts as therapeutic strategies since their pharmacological properties and chemical composition have been thoroughly assessed by several studies [10]. Although more work is required to understand the full extent of its therapeutic properties, the research conducted so far has attributed UD to several health benefits. For instance, the leaves of the nettle are highly rich in flavonoids, phenolic acids amino acids, saturated fatty acids, carotenoids, and organic acids (Figure 1) [11,12,13,14,15,16]. Previous research established that flavonoids, including rutin, luteolin, and quercetin, found in UD, possess anti-inflammatory and analgesic properties [17,18,19]. The former benefits auto-immune inflammatory disorders such as rheumatoid arthritis, rhinitis, and rheumatism, whereas the latter can limit oxidative damage, an important aspect of organ toxicity, malignant proliferation, and cancer progression [20]. Several bioactive molecules, including organic acids, namely formic, succinic, malic, and quinic acids, were shown to concentrate in the aerial organs of the plant, specifically the leaves more so than the stem or stalks [21,22]. Phytochemical analysis of the nettle leaves reveals the presence of several phenolic acid compounds: hydroxybenzoic acid derivatives (e.g., gallic, vanillic, syringic, protocatechuic, and gentisic acids) and cinnamic acid derivatives (e.g., cinnamic, caffeic, coumaric, ferulic, chlorogenic, and sinapic acids) [23]. As for the roots, their extracts contain fatty acids (polyunsaturated and monounsaturated fatty acids), lectins, sterols, polysaccharides, and lignans, which have been shown to improve the symptoms of benign prostatic hyperplasia, inhibit the proliferation and migration of cancer cells, and possess anti-angiogenic properties [5,16,24] (Figure 1). Even the seed oil of the nettle plant has been shown to carry antioxidative compounds such as fatty acids (linoleic and linolenic acid) and sterols such as β-sitosterol [25,26]. Interestingly, symbiotic endophytes such as *Bacillus cereus* or *Bacillus mycoides*, isolated from UD, have been identified as reservoirs of secondary metabolites, particularly polyphenols, with properties mirroring those of UD itself [27]. As a green plant capable of photosynthesis, its leaves and seed oil also contain chlorophyll and carotenoids such as β-carotene, violaxanthin, and xanthophylls, whose abundance is significantly influenced by the time of harvest [5,8,21,25,28,29]. Nettles have long been studied for their nutritional values, particularly for their substantially higher quantities of protein content relative to wheat and barley flours, as reported by a study conducted by Adhikari et al. (2015) [30]. The higher content of polyphenols contributes to the cleansing of the digestive system, improvement of bad breath and bloating, and detoxification of the liver [5]. Hence, from a nutritional standpoint, the leaves of UD are of great value due to their richness in water-soluble vitamins (vitamins B and C) and fat-soluble vitamins (vitamins A, D, E, and K) as well as their mineral composition including iron, calcium, magnesium, zinc, and soluble silica [8,10,19,31,32]. In fact, their introduction into animal diets has improved growth metrics and blood cholesterol levels [33,34]. Upon feeding nettle as forage to pregnant ewes, increased milk production and lamb growth along with a decreased rate of disease were observed [35,36]. The availability and preparation of UD for consumption were also investigated, revealing that the freeze-drying method results in better magnesium, phenolic, and antioxidant content when compared to convective methods [35]. However, amongst conventional forms of heat drying, convective or micro-wave drying of UD leaves displays distinctly lower energy consumption and higher color retention compared to other methods, such as vacuum drying [37,38]. Moreover, UD has also been reported to affect various other bodily function mechanisms, including reducing cancer symptoms, wound healing, cell repair, and the control of cardiovascular disorders [5,18,39,40]. It is also worthy of mention that the nettle plant can be used as a potential defense against pathogenic microorganisms, namely Gram-negative and Gram-positive bacteria [41].

Given all of these benefits and the eventual rise in demand for plant-derived therapeutic compounds for experimentation or consumption, the optimization of UD cultivation to prioritize yield and availability of bioactive molecules is incentivized. To that end, in terms of agronomical factors, proper weed management, uniform irrigation practices, and a supply of organic amendments for the soil were shown to be associated with a successful harvest [42]. Topographically, UD’s distribution extends across both lowlands and mountainous terrains, with a propensity for partially shaded areas. In parallel, fertile damp soil rich in nitrogen, whether weakly acidic or weakly basic, can accommodate the growth of UD, e.g., clay loam soil composition [21,42]. As for the harvest, the vegetation period, prior to flowering, remains optimal for the collection of UD leaves for therapeutic applications. Conversely, Paulauskienė et al. (2021) have suggested that variability in antioxidant capacity or composition between plants harvested at different stages of the vegetation period can be attributed to differences in meteorological conditions, including rainfall, temperature, and sunlight exposure [8,21]. Further, roots harvested in autumn, at the end of the vegetation period, have been shown to have a higher bioactive molecule content [42,43], while a positive relationship between increased exposure to sunlight and the phenolic content of individual UD plants has been shown [22]. Consequently, regions meeting these criteria produced UD harvests that exceeded others with respect to biomass, polyphenol content, and ascorbate levels [42]. Transitioning to the extraction process, brewing water extracts of UD at higher temperatures has been shown to elevate the antioxidant capacity of the final product [43], in contrast to ultrasound-assisted enzymatic extraction showing diminishing returns after raising the temperature past 60 °C [11]. In terms of solvents, alcohols, including methanol or ethanol, and water have been used in various combinations to maximize the retention of UD’s beneficial properties for therapeutic potential [11,12,13,14,20,43,44].

The purpose of this review is to provide a comprehensive and up-to-date assessment of the various therapeutic effects of *Urtica dioica* by reviewing its possible anticancer, antidiabetic, anti-inflammatory, antimicrobial, and analgesic properties as well as its systemic effects on the nervous, cardiovascular, reproductive, digestive, and excretory systems to contextualize various medical conditions for which the plant may be of use. Some reviews have focused on the promising antidiabetic potential [45], antioxidant properties [46], neuroprotective effects [47], and anti-tumorigenic role in breast cancer [48], in addition to the food functional properties of UD [23]. The body of literature in this review will be referenced in order to assimilate the leading hypotheses for the various effects of UD, reporting data from in vivo, in vitro, and clinical trials where applicable in order to identify the gaps necessitating further experimentation or clinical trials.

## 2. Anticancer Effect

**Figure 2:**
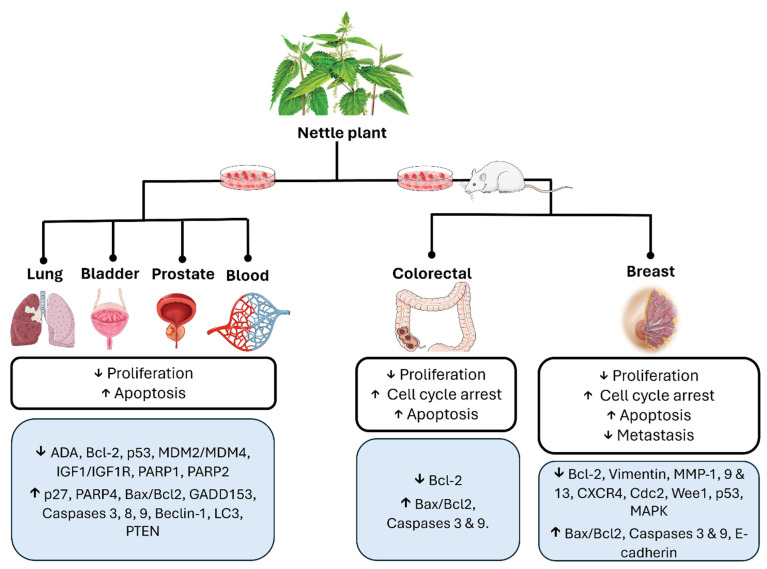
Overview of the anticancer properties of the nettle plant (↑: increase ↓: decrease).

In the past two decades, extensive studies reported the therapeutic potential of UD leaf and root extracts for the treatment of various types of cancers, with a major focus on breast tumor malignancy [48]. The following sections, summarized in Figure 2, assess the major developments in this area, whether in vitro or in vivo and highlight the gaps that require further investigation. 

### 2.1. Prostate Cancer

Prostate cancer was the second most common malignancy in males globally with an increasing percentage of prostate cancer deaths worldwide in the 21st century [49]. A variety of therapeutic techniques were used for treatments, including traditional chemotherapy, radiation therapy, and surgical removal. However, the high mortality rate demonstrates the need to look into other therapeutic strategies. UD is one of various medicinal plants with therapeutic promise due to its proapoptotic activity in cancer cells [50]. The anticancer effects of the nettle plant on several prostate cancer cell lines have been examined as an alternative approach to traditional cancer treatments.

Different types of UD extracts including dichloromethane, methanolic, and aqueous extracts were evaluated and found to exhibit a cytotoxic effect on several prostate cancer cell lines, namely PC3 and LNCaP, in a dose- and time-dependent manner [50,51,52]. A differential response was observed on different prostate cancer cell lines depending on the hormonal sensitivity of each. In one study, researchers reported that DTH (3,4-divanillyltetrahydrofuran) extract from UD roots was more cytotoxic to LNCaP cells than PC3 cells due to their differences in androgen sensitivity [53]. UD was also shown to inhibit the proliferation of PC3 prostate cancer cells by inducing a G2/M cell cycle arrest, hence halting the cell cycle progression at a critical checkpoint during cell division [50].

Another mechanism for the anticancer effects of UD is the activation of the programmed apoptotic cell death mechanism. The molecular mechanisms through which UD extracts exhibit their anticancer activity range from reactive oxygen species (ROS) production, mitochondrial dysfunction, and DNA damage to proapoptotic caspase cleavage. UD extracts were proven to promote DNA fragmentation, one of the most fundamental aspects of apoptosis, in both PC3 and LNCaP cancer cells [50,51]. Additionally, studies indicated that UD extract activates the cleavage of poly ADP ribose polymerase (PARP), a protein involved in DNA repair, in the aforementioned prostate cancer cells [51,54]. Another hallmark of apoptosis is mitochondrial depolarization, which is characterized by increased permeability of the mitochondrial membrane and the release of cytochrome c, a caspase-9 activator, into the cytosol [55]. Levy and colleagues revealed in their study the ability of UD to decrease mitochondrial membrane potential in prostate cancer cells. Along with this, the authors confirmed increased caspase-3 and caspase-9 activity upon UD exposure, providing more evidence for the role of apoptosis in prostate cancer cell death [51], similar to another study that demonstrated an upregulated mRNA expression for both caspase 3 and caspase 9 in PC3 cells treated with UD [50]. Moreover, UD promoted inhibitory effects on the expression of the antiapoptotic marker Bcl-2, a protein that reduces the permeability of the mitochondrial membrane and inhibits the release of cytochrome c [50,56]. Lastly, the current literature indicates that there is a link between decreased mitochondrial membrane potential, ROS production, and apoptosis in cancer cells [57,58]. In the study conducted by Levy et al., the authors concluded that apoptosis activation, revealed by an increase in apoptotic markers along with DNA fragmentation and membrane depolarization, could be due to the accumulation of ROS in these cells upon exposure to UD as compared to the control group [51].

To the best of our knowledge, no in vivo studies have been reported in the literature evaluating the anticancer properties of UD on prostate cancer despite the extensive in vitro studies conducted. One ex vivo study, performed by Durak et al., assessed the ability of UD to halt prostate cancer progression in prostate cancer tissue extracted from patients; the authors noted a significant inhibition of the adenosine deaminase (ADA) enzyme, involved in the elimination of the toxic deoxyinosine molecule. As such, another mechanism that can explain the anticancer activity of UD is promoting cancer cell death via the accumulation of toxic metabolites [59]. Despite the promising data obtained from in vitro studies, further confirmation of the effect of UD in prostate cancer is needed from in vivo and clinical trials.

### 2.2. Breast Cancer

Several studies have revealed that UD exhibits antiproliferative properties along with proapoptotic effects on many breast cancer cell lines, including MCF-7 and MDA-MB cells. To analyze and evaluate those properties, Fattahi et al. reported a dose-dependent antiproliferative activity of UD extract on an MCF-7 breast cancer cell line along with a prominent antioxidant activity of powdered UD [60], which might be attributed to its flavonoid content, such as kaempferol, quercetin, and rutin content [61]. In the same study, the authors also elucidated the ability of UD to suppress MCF-7 cell proliferation via the activation of the apoptotic pathway: the mechanism was promoted via caspase-9 activation through calcium overload, which in turn triggers cytochrome c release from the mitochondria. As such, and through caspase 3, apoptosis is initiated by protein cleavage and membrane blebbing along with DNA fragmentation [60]. Similarly, UD was also found to promote the activation of apoptosis in MCF-7 cells upon increasing the levels of apoptotic markers, particularly the Bax/Bcl2 ratio [62]. Alternatively, Wenyua et al. (2021) suggested that by inhibiting the phosphorylation of PI3K/AKT pathway elements, UD is able to inhibit cell proliferation, decrease cancer cell viability, and induce cell cycle arrest at the G0/G1 phase, hence promoting apoptosis [63]. Additionally, the reduced expression of Ki-67 and increased expression of p53, which are regarded as a proliferation marker and a cell cycle regulator, respectively, have also been documented in breast cancer cells [64]. Recently, Upreti et al. (2023) evaluated the anticancer potential of UD on breast cancer cells in silico. Their results revealed a significant binding affinity of UD ligands with JAK2, which was then confirmed in vitro, confirming the selective cytotoxic effect of UD to be via the JAK2/STAT3 pathway, a constitutively expressed pathway in TNBCs [65].

From a different aspect, the inhibitory effect of UD on breast cancer cell motility was also assessed. UD was reported to have a selective and considerable ability to reduce the proliferation as well as the migration of MCF-7 cells while having no effect on an HFFF2 normal cell line [66]. The results are in line with another study that confirmed the efficacy of UD in inhibiting MCF-7 and MDA-MB-231 breast cancer cell migration. This was revealed by the downregulated expression of miR-21 genes; matrix metalloproteinases (MMP)-1, 9, and 13; E-cadherin; vimentin; and CXCR4, all of which play a crucial role in promoting cell metastasis. As such, the authors concluded that UD extract has the ability to halt the metastatic properties of breast cancer cells [66].

As for aggressive breast cancer cells, notably triple-negative MDA-MB cells, a number of studies provide significant data that could potentially deliver promising results for the role of the nettle plant in combination with chemotherapeutic drugs, a novel approach for better outcomes on patients’ health. Mohammadi et al. aimed to evaluate the potential use of UD with paclitaxel drug on triple-negative breast cancer (TNBC) cells, revealing its ability to enhance the sensitivity of MDA-MB to paclitaxel treatment. The authors suggested that this combination might promote cancer cell death via cell cycle arrest at the G2/M phase along with a downregulation of Cdc2 and wee1 expression, key regulatory factors of the cell cycle progression. Moreover, this combination had an inhibitory effect on the migration of the MDA-MB-468 cell line via the inhibition of Snail-1 gene expression, which is correlated with cell migration and metastasis [67]. These notions are in line with our own findings whereby a leaf infusion of UD similarly sensitized MDA-MB-231 cells to the chemotherapeutic drug, cisplatin, resulting in a dose- and time-dependent antiproliferative effect. The observed cell death was confirmed to occur via the activation of the intrinsic apoptotic pathway as revealed by the increase in the Bax/Bcl-2 ratio, DNA fragmentation, and PARP cleavage [68].

It is noteworthy that nanoparticles have emerged as drug delivery systems with a deeper reach than traditional chemotherapies. Their increased efficiency is also thought to reduce the reliance on larger dosages, with minimal toxicity on normal cells [69]. This is best exemplified in the recent work of Daglıoglu et al. (2023), where silver nanoparticles synthesized from UD leaf extracts exhibited dose-dependent antiproliferative and apoptotic effects on MCF-7 cells that far exceeded those of the extract alone [70], similarly to the selenium-based nanoparticles on a HepG2 cancerous cell line [69]. All of these findings combined emphasize the role of UD in the manufacturing of nanoparticles, with the aim of treating breast cancer among others. 

Given the extensive basic studies conducted, the benefits of the nettle plant against breast cancer were also put into experimentation in in vivo studies. Using animal models and upon injection with UD extracts, tumor development and volume were found to be substantially decreased [64]; this is in line with the results obtained from the consumption of aqueous UD extract, which caused a lower incidence of breast cancer in mammary tumors generated in rats [71]. In another study, the authors reported the anticancer activity of UD on a BALB/c mouse model of breast cancer. Their data displayed a prominent decrease in tumor mass and size in the treated group, which, according to the authors, is due to the activation of apoptosis [72]. Translating these in vitro and in vivo studies into clinical trials is needed to further confirm the anticancer properties of the nettle plant and improve breast cancer patients’ health. 

### 2.3. Blood Cancers (Leukemia)

Leukemia is a blood cancer that arises in the bone marrow and substantially leads to an abnormal leukocyte blood count [73]. Despite the available treatments like chemotherapy and radiation therapy, the need for novel and effective therapeutic strategies is evident. UD was shown to exhibit an antiproliferative effect on various leukemic cell lines. For instance, UD leaf extract demonstrated a significant inhibitory effect on the growth of HL-60, U937, and KG-1 cells in vitro [74,75,76] similar to the effect of UD agglutinin on Jurkat and Raji cells [77]. The selectivity of UD was further demonstrated by studies conducted on normal human B lymphocytes in our lab and PBMC cells treated under similar conditions, which revealed no significant cytotoxic effects [75,78]. Consequently, it could be deduced that UD might be a prominent and safe anticancer agent to be used for the treatment of leukemia. 

A possible mechanism of action for UD is targeting the cell cycle progression. In fact, UD extract caused a time- and dose-dependent increase in pre-G0 content in U937 cells [75], while displaying an increase in the sub-G1 phase in HL-60, Jurkat, and Raji cells [74,77]. Along with these results, exposure to UD extracts resulted in a remarkable downregulation of genes involved in the cell cycle pathway, such as the MDM2 gene [78]. 

Hallmarks of apoptosis were further investigated to elucidate the mechanism by which UD disrupts the proliferation of leukemic cells. Flow cytometry results from various leukemia cell lines treated with UD showed a significant increase in Annexin V binding to phosphatidylserine upon the translocation of the latter to the outer leaflet during apoptosis [74,75,77,78]. Along these lines, our lab quantified DNA fragmentation and reported a significant increase in the enrichment of oligo-nucleosomal fragments in U937 cells [75]; activation of the mitochondrial-dependent pathway of apoptosis was further demonstrated by the significant increase in the Bax/Bcl-2 ratio. Moreover, the mitochondrial membrane potential, a key indicator of healthy intact mitochondria, was found to be responsible for promoting cell death in HL-60 cells [74]. Disruption in this potential could have detrimental outcomes and commit the cells to the apoptotic pathway. Other crucial regulators of apoptosis are PARP genes involved in DNA repair, particularly PARP2, which was downregulated in HL-60 cells exposed to UD extract, thereby promoting the activation of apoptosis. On the other hand, it should be noted that PARP4 can exhibit opposing effects. Interestingly, agglutinin from UD was reported to inhibit HL-60 cell growth via the upregulation of PARP4 expression [78]. The involvement of both extrinsic and intrinsic pathways was put into evidence by the activation of caspases 3, 8, and 9 along with an increase in the Bax/Bcl-2 ratio and other proteins such as p53 and p27 [74,77]. Moreover, UD extract could exhibit cytotoxic effects on leukemic cells by targeting various cellular mechanisms such as the IGF1/IGF1R signaling pathway, known to be a key factor in the progression of many cancers, especially acute myeloid leukemia [78]. UD agglutinin extract was also found to significantly promote apoptosis in Jurkat cells while exhibiting a non-significant effect in Raji cells. This resistance to apoptosis was examined by Kawabata et al. (1999), reporting a lack of caspase activity [79]. Additionally, Luciano et al. (2002) corroborated this finding by detecting a low ratio of DFF40 to DFF45, signaling the absence of DNA fragmentation [80]. Further studies should elucidate the mechanism behind this resistance while also conducting further experimentation on animal models and in clinical trials to better understand the ability of UD to halt the progression of leukemia. 

### 2.4. Colorectal Cancer

The prevalence of colorectal cancer, known to be a common type of digestive malignancy, is still on the rise despite the universal use of conventional treatments, pushing the scientific community to thoroughly investigate alternative approaches [81]. 

Most of the literature targeted the human colon carcinoma HT29, colorectal carcinoma HCT116, and the colon adenocarcinoma Caco-2 cell lines to investigate the anticancer properties of UD. Nettle plant extract was shown to significantly inhibit the proliferation of these cell lines in a dose- and time-dependent manner, with minimal effects against healthy human colorectal cells (HFF) [81,82,83], highlighting its promising potential use amongst other treatments. Furthermore, there is compelling evidence that oxidative stress is a mediator of apoptosis [84,85]. With that in mind, UD, through the mediation of lipid peroxidation, has been reported to increase ROS content, promoting oxidative stress [86,87]. At the same time, other studies have documented cell cycle arrest at the G2 phase and DNA fragmentation upon UD treatment [81]. Combined, these aspects suggest the ability of UD to induce an apoptotic response in colorectal cancer cells. To support this conclusion, Kardan et al. (2020) highlighted the selective cytotoxic effect against healthy HDF epidermal cells [83]. As an extension, following UD treatments, the mRNA and protein expression of Caspases 3 and 9, as crucial markers of apoptosis, was increased, the mRNA expression of the antiapoptotic Bcl-2 protein was decreased, and the ratio of Bax/Bcl-2 was elevated [81,83,88]. However, Ghasemi et al. (2016) recognized that the precise signaling pathway behind such results remains elusive given the diversity of compounds within *Urtica dioica* extracts, including isolectins, phenols, and triterpenoic acids [86].

As for other properties, UD has been documented to exhibit chemo-preventive effects when supplied simultaneously with a tumor inducer in animal models. Specifically, UD supplementation reduced colon cancer incidence rate and development, restored the activities of several antioxidant enzymes, and induced apoptosis through elevated Caspase 3 expression [88]. In addition, advancements in nanotechnology, namely nanoemulsion and nanoencapsulation, have risen to prominence in the medical field due to their increased bioavailability, controlled release, and increased specificity. As it relates to colon cancer, both UD nanoemulsion and nanoencapsulated UD extract showed increased antiproliferative effects against HCT-116 as compared to a hydroethanolic extract [89]. In light of the established basic research studies, UD extracts might be a promising option for additional clinical research and alternative therapies.

### 2.5. Other Types of Cancer 

The promising anti-tumor effects of UD on other types of cancer, including cervical, gastric, and lung cancer, have also been reported in the literature [48,86,90]. However, combination therapies have been recently extensively investigated; the detrimental side effects of chemotherapeutic agents have resulted in a strong incentive to find novel ways to increase the efficiency of these treatments without resorting to higher dosages. One such method is the reliance on combination therapies utilizing natural compounds. Two types of cancers that have been investigated in this respect are bladder cancers and non-small cell lung carcinoma (NSCLC). The combination of an N-butanol UD extract and doxorubicin, a drug used in the treatment of bladder cancer, among other types, exceeded the apoptotic effect of each drug alone on T24 bladder cancer cells [91]. In the case of NSCLC, which responded poorly to cisplatin treatment, UD displayed a selective antiproliferative effect with no harmful effect on normal cells. Interestingly, co-treatment exhibited a synergistic anticancer effect by promoting the activation of apoptosis via the extrinsic pathway in addition to an arrest in the cell cycle at the G2/M phase [92]. This is in line with the promising results of combination therapies that have been reported previously in breast cancer cells, as elaborated above. Moreover, the combination of UD extracts with other medicinal plants has also yielded promising results. Specifically, Rahmani et al. recently showed that the combination of UD and Wormwood (*Artemisia absinthium*), as a hydroethanolic extract, had a more prominent anticancer effect on HCT-116 cells compared to each alone [89]. Given the extensive body of literature and studies demonstrating the anticancer properties of UD extracts on various cancer types (Table 2), the nettle plant provides a promising chance for its use in cancer prevention and treatment to improve the survival rate of patients.

## 3. Antidiabetic Effect

Diabetes is a severe metabolic illness that affects people from all backgrounds of life, regardless of geography, ethnicity, or race, and its incidence and prevalence are increasing at an alarming pace internationally [93]. Ethnobotanical data suggest that over 800 plant species, including UD, are believed to possess antidiabetic properties, as reported in many in vivo studies [94,95,96].

A study conducted by Patel and Udyabanu (2014) investigated the antidiabetic properties of a hydro-alcoholic extract of UD on dexamethasone-induced diabetic mice. Their data demonstrated that UD effectively normalized elevated blood glucose levels, reduced body weight, and attenuated increased water intake induced by Dexamethasone exposure, a glucocorticoid receptor agonist [97,98]. Congruent results were noted to ascertain the antidiabetic effect on Streptozotocin (STZ)-induced type 2 diabetic mice based on weight loss prevention and improvements in fasting blood glucose and insulin levels [99,100]. Several studies further elaborated on the UD extract mechanism of action, indicating that it increases β-cell insulin secretion and hepatic glucokinase, hexokinase, and glucose-6-phosphate dehydrogenase enzymes [100,101,102]. Similarly, Pérez Gutiérrez et al., who recently investigated the effects of the orally administered extract combination of UD comprising *Apium graveolens* and *Zingiber officinale* (UAZ) on diabetic mice, reported an improvement in hepatic enzymes and a reduction in liver weight, which reveals the hypolipidemic and hypoglycemic effects of UD [103]. 

Interestingly, a study conducted on type 2 diabetes mellitus patients reported that UD can decrease the risk of cardiovascular diseases as they observed an increase in high-density lipoprotein (HDL) [104]. Furthermore, Fan et al. (2020) corroborated the protective role of UD in preventing insulin resistance in high-fat-diet mice (HFM) via significantly reducing adipocyte fat accumulation [105]. Moreover, HFM mice treated with UD exhibited lower levels of CD11c, an inflammatory marker associated with insulin resistance, compared to low-fat-diet mice, while it significantly increased the fasting-induced adipocyte factor, which reduces the ability to store fatty acids in peripheral tissues.

Chronic hyperglycemia itself can dysregulate many of the body’s vital processes, potentially leading to cardiomyopathy, which is characterized by an increase in PGC-1α and NRF2, key regulators of cardiac mitochondrial biogenesis. As such, Seyyedeh et al. recently aimed to elucidate the impact of UD supplementation, in conjunction with an endurance training protocol, on various metrics of cardiac function in STZ-induced diabetic mice [106]. The results revealed the ability of the extract to increase ATP content, citrate synthase activity, and the expression of PGC-1α as well as that of NRF2. Additionally, Mehrezi et al. discovered that UD extracts can also increase antioxidative enzymes, such as superoxide dismutase, while reducing oxidative stress biomarkers, including thiobarbituric reactive species and lipid hydroperoxides [104]. Furthermore, recent investigations highlighted the neutralizing effect of UD bioactive compounds on ROS production in insulin-secreting β-cells, thereby preventing oxidative stress-induced diabetes [107]. In vitro studies reported the inhibitory effect of UD on the enzymatic activity of α-amylase and α-glucosidase, thus reducing glycemia [108]. This is in line with the data of another study conducted by Rahimzadeh et al., whereby UD promoted a noticeable inhibition of maltase, sucrase, and lactase activities [109].

In the case of insulin resistance, muscle and adipose are unable to translocate GLUT4, a facilitative hexose transporter, to the cellular surface, contributing to an aberrant glucose homeostasis [110]. The exposure to UD extract increased GLUT4 translocation to the cell surface compared to the control group, reaffirming the antidiabetic properties of UD. This aligns with the previous work reporting the effect of UD extract in reversing the decrease in Hippocampal GLUT4 mRNA expression [97].

Interestingly, the antidiabetic potential of UD has further been confirmed in several clinical trials in patients suffering from type 2 diabetes [111]. Nettle leaf extract was effective in lowering blood levels of fasting glucose and glycosylated hemoglobin (HbA1c) in patients with type 2 diabetes as compared to a placebo group, with no effect on creatinine, liver enzymes, or blood pressure [112]. Along with this, a recent investigation also reported a positive effect of UD extract on type 1 diabetes; the intervention group had lower blood glucose and total insulin levels, which further expands the antidiabetic properties of UD [113,114]. 

## 4. Antimicrobial Effect

The use of natural plant-derived antimicrobial compounds for food preservation has become crucial. The overuse of antibiotics and antifungal drugs has adverse health reactions, which might lead to drug resistance [115,116,117]. Furthermore, viral mutations have rendered many of the traditional antiviral drugs and vaccines less effective [118]. For these reasons, the use of natural plant-derived extracts such as UD has become increasingly significant in treating bacterial, fungal, and viral infections [116].

Several studies were conducted to investigate the potential antibacterial effects of UD on inhibiting the growth of Gram-negative and Gram-positive bacteria, suggesting its promising effect in pharmaceutical as well as food industries [69,119]. It was also reported that UD methanolic extract had a considerable antibacterial effect against methicillin-resistant *Staphylococcus aureus* (MRSA) isolates [120]. This was further demonstrated by the topical application of ointment made from UD to accelerate the wound healing process by preventing bacterial colonization by MRSA [121]. In addition, when UD was used in wound dressings along with zinc oxide, medical scaffolds became much stronger against *Staphylococcus aureus* and *Escherichia coli* [122]. One study revealed that the essence of UD leaves significantly inhibited the growth of *Klebsiella pneumoniae* and *Bacillus cereus*. However, the essential oils only exhibited an average inhibitory activity on *Staphylococcus aureus* and *Pseudomonas aeruginosa* [123]. Numerous other studies have also confirmed the antibacterial properties of UD on other bacterial species including *Bacillus subtilis*, *Shigella dysenteriae*, *Salmonella typhi*, *Pseudomonas fragi*, *Campylobacter jejuni*, *and Listeria monocytogenes* [69,115,124,125,126,127]. It is important to note that UD extract did not cause any adverse effects on non-pathogenic bacteria such as the beneficial bacteria Lacticaseibacillus and Bifidobacterium [128]. In addition, UD was reported to work synergistically with other drugs and natural compounds such as tetracycline, erythromycin, and chloramphenicol by decreasing the minimum inhibitory concentration (MIC) of these drugs in the treatment of *Escherichia coli*, *Staphylococcus aureus*, and *Klebsiella pneumoniae* [129]. The molecular methods by which UD exerts its antibacterial properties have also been investigated, suggesting its ability to disrupt bacterial cell structure and phosphorus metabolism, leading to cellular material leakage into the environment, hence promoting bacterial death [116].

Looking further into the antimicrobial properties of the nettle plant, many studies have explored its antifungal and antiviral activity. In one study, selenium nanoparticles (SeNP) synthesized using UD revealed antifungal properties against unicellular and multicellular fungi including *Candida albicans*, *Aspergillus fumigatus*, *Aspergillus niger*, *Aspergillus flavus*, and *Candida lipolytica* [69,115,129]. In a similar investigation, researchers reported the significant antifungal effect of UD against *Candida albicans* and *Candida parapsilosis* despite the stronger effect of the antifungal drug amphotericin B [117]. Furthermore, the antiviral effect of UD was assessed against the SARS-CoV-2 virus, confirming its inhibition of virus entry, replication, and infectivity [130,131,132]. The inhibitory effect was found to be due to the ability of UD to block angiotensin-converting enzyme 2 (ACE2), a receptor that is required for the attachment and entry of the SARS-CoV-2 and Rabies viruses into the cell [118,133].

Interesting research on UD’s antimicrobial and antiparasitic benefits was evaluated using in vivo models. A study conducted by Badirzadeh et al. reported that nettle plant extract can decrease the number of parasites in cutaneous leishmaniasis, an opportunistic disease in HIV-infected individuals. It is also important to note that interferon (IFN)-gamma levels were increased after injection with the extract, while the number of macrophages remained the same. The study shows the effectiveness of UD in targeting parasitic infections in mice while also being harmless to macrophages and inducing beneficial immune mechanisms [134]. Along with this, another study focused on the effect of UD on mice with toxoplasmosis, a parasitic infection caused by Toxoplasma gondii. In this study, UD-treated mice had a higher survival rate, decreased number of brain cysts, and increased levels of INF- γ [135]. These results together, summarized in Table 3, confirm the antimicrobial properties of UD against bacterial, viral, and fungal infections.

## 5. Anti-Inflammatory Effect

Various studies investigated the promising effect of UD on treating inflammatory conditions due to its flavonoids and phenolic acids content, which can lower the synthesis of inflammatory mediators [136]. Hence, UD has been utilized in world traditional medicine for treating anemia, dermatitis, and joint pain for hundreds of years [90].

Tumor necrosis factor (TNF) levels are elevated in numerous inflammatory diseases and may be responsible for the increased expression of some proinflammatory genes. In vitro studies reported the effect of nettle leaf extract in inhibiting TNF-kappa activation in several cell types, namely human T lymphocytes, macrophages, and epithelial cells [137]. Nettle extracts were also evaluated for their ability to reduce the levels of Interleukin (IL)-1β-induced Nuclear Factor Kappa Beta (NF-κB). NF-κB is a transcription factor that enters the nucleus and stimulates the production of proinflammatory and proapoptotic genes. The extracts of nettle were able to suppress the NF-κB signaling pathway and block the translocation of activated NF-κB to the nucleus in chondrocytes [138]. The anti-inflammatory effect of UD was also investigated using macrophage immune cells (RAW264.7); it was reported that methanolic extract preparation from the flowering part of the nettle plant promotes a prominent anti-inflammatory effect with minimal cytotoxicity when compared to other extract preparations from different plant parts [139]. Another study showed that stinging nettle also exhibits anti-inflammatory properties by blocking the enzymes cyclooxygenase and lipoxygenase, which produce the proinflammatory mediators prostaglandins and leukotrienes, respectively. Their data also demonstrated that an alcoholic UD extract can lower the concentrations of TNF-α and IL-l, two proinflammatory cytokines whose release in the blood is induced by lipopolysaccharides [140]. These findings have been translated into clinical studies that revealed the anti-inflammatory effect of UD in patients with inflammatory bowel disease and rheumatoid arthritis [141,142]. Moreover, a double-blind clinical trial reported no major effects of UD root extract in reducing inflammation of allergic reactions as compared to the placebo; however, the authors underscored the major limitation of their findings being the limited sample size in addition to the need for longer-term studies [143].

## 6. Analgesic Effect

Despite the pain relief offered by opioids, non-steroidal anti-inflammatory medicines (NSAIDs), antispasmodics, calcium channel blockers, and antihistamines [144], the negative side effects associated with their long-term use remain a point of contention. In this aspect, the available data present UD as a reasonable alternative, whereby it has been reported that UD exhibits analgesic effects in acetic acid-induced writhing and formalin tests, with the former widely employed for analgesic drug screening [136,144]. As for the latter, UD was shown to reduce the pain response in the phase correlated with an inflammatory response, suggesting that the observed analgesia stems from the previously described anti-inflammatory action of UD [145]. In accordance, an aqueous extract of nettle leaves and stems conferred resistance to thermal stimulation in mice models. Together, these findings point to analgesic activity in the peripheral nervous system partly due to the presence of flavonoids, caffeoyl malic acid, and caffeic acid among others [31,145]. On the other hand, nettle leaves, when applied externally, possess similar analgesic properties. In particular, participants in a study conducted by Randall et al. (1999) unanimously reported improvements in their joint discomfort/pain after applying fresh UD leaves to their body [146]. A following randomized control trial revealed similar reductions in wrist pain following UD application to the area of interest [146].

## 7. Systemic Effects

**Figure 3:**
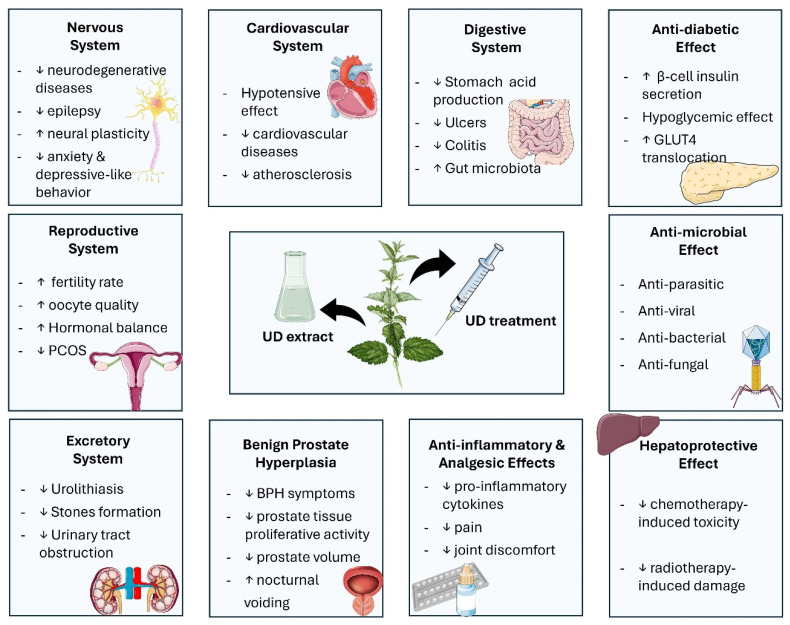
Overview of the nettle plant’s antidiabetic, microbial, and inflammatory properties and protective effects on the liver, reproductive, excretory, cardiovascular, nervous, and digestive systems (↑: increase, ↓: decrease).

In addition to the various therapeutic effects detailed above, the literature further confirms the protective effects of UD on various organ systems in the human body, which are all summarized in Figure 3 and further elaborated in the following sections.

### 7.1. Hepatoprotective and Anti-Toxic Effects of UD

The liver is the second-largest solid organ and one of the most important organs of the human body for the regulation of homeostasis. It eliminates contaminants and other toxins from the body’s blood supply, controls blood coagulation, and performs numerous other vital functions [147]. Aside from the numerous synthetic medications that are accessible, several forms of natural and herbal remedies derived from plants, including UD with its phytoconstituents, are used to treat liver illnesses. For instance, a large dose of cisplatin, a chemotherapeutic agent, has been linked to nephrotoxicity and hepatotoxicity. As a consequence, Ozkol et al. (2012) investigated the hepatoprotective effects of UD methanolic extract against cisplatin toxicity in Erhlich ascites tumor (EAT)-bearing mice. They demonstrated the protective effect of this extract of enhancing the antioxidative defense systems and minimizing the deleterious impacts on the liver [148]. The same hepatoprotective effect extends to other harmful compounds, namely ethylene glycol, previously shown to damage hepatic tissue. Interestingly, UD was shown to minimize inflammatory cell infiltration as part of its improvement of hepatic histopathology [149]. Moreover, scientists recently aimed to investigate the radioactive protective activity of UD seed extract in the whole blood and liver of radiation-administered rats. The radiation group exhibited an increase in the levels of 8-hydroxy-2-deoxyguanosine (8-OHdG), a biomarker for endogenous oxidative DNA damage [150], and a reduction in Gpx-1 immunoreactivity, which prevents ROS production. However, an amelioration in those parameters was observed in the radiation group treated with UD [151,152]. In parallel, Yıldızhan et al. concluded that UD prevented radiotherapy-induced liver damage as well as lipid peroxidation and oxidative stress, in addition to the protection of antioxidant enzymes. Overall, these results highlight the potential role of UD as a radioactive protector [153]. Furthermore, the injection of rats with UD resulted in a significant choleretic effect, the stimulation of bile secretion from the liver, the synthesis and excretion of cholates, and, finally, bilirubin and cholesterol excretion [154].

Heavy metals, if found in high amounts in the soil, tend to bioaccumulate in vegetables, which are eventually consumed by animals and humans. Even though metals like copper and zinc are essential nutrients for humans, at high concentrations, they might cause numerous diseases, including cardiovascular diseases, arteriosclerosis, gastrointestinal immunity disorders, and even neurodegenerative illnesses [155]. Multiple studies investigated the beneficial effects of UD, particularly on heavy metal toxicity [156]. Recently, an in vivo study on copper sulfate-poisoned rats treated with UD reported a significant decrease in kidney weight, glucose, uric acid, urea, creatinine, triglycerides, and LDL alongside a significant increase in body weight and HDL, with no significant alterations in cholesterol and VLDL levels [157].

From a different aspect, inflammation of pancreatic and peripancreatic tissues can be partly traced back to the secretion of inflammatory cytokines or the release of free radicals, which both induce hepatocyte apoptosis. In this respect, Yılmaz et al. demonstrated that UD improved edema, pancreatic necrosis, and pancreatic inflammation in a TNF-α-independent manner while reducing apoptosis [158]. In conclusion, these studies incorporate UD’s various aforementioned modes of action in toxicity prevention and hepatoprotection.

### 7.2. Effect on the Nervous System

The presence of the blood–brain barrier, the interconnectivity of different molecular pathways, and the risk of unintended side effects arising in the same or distant structures constitute some of the limiting factors halting the progress in the treatment of CNS diseases [159,160]. Hence, alternative sources of bioactive molecules that can circumvent these criteria are a valuable substitute for traditional medications [161,162]. *Urtica dioica* contains various compounds, including scopoletin, 5-hydroxy tryptamine (5-HT), and carvacrol [34], which were reported to promote potentiation within the hippocampus, contribute to memory formation, and possess neuroprotective properties, respectively [163]. Consequently, this positions UD as a prime candidate for the treatment of diabetes-induced neuropathy, cognitive decline, neurodegenerative diseases, or depression, leading to the successful clinical studies that have been recently reviewed by Semwal et al. [47]. 

Fundamentally, inducing type 2 diabetes is expected to detrimentally alter both insulin signaling pathways and ROS levels in the hippocampus, contributing to a depressive-like phenotype in mice models [97,99]. Interestingly, it was shown that UD treatment improved insulin signaling through the mediation of insulin receptor and insulin-like growth factor-1 receptor (IGF1R) levels, reduced oxidative stress, and opposed neuroinflammation and apoptosis [99]. To be specific, UD treatments improved various biomarkers including malondialdehyde (MDA), thiobarbituric acid-reactant substances (TBARS), superoxide dismutase (SOD), and catalase activity, highlighting a potential neuroprotective role for UD in combatting neuronal cell death [97,99,164]. Recent studies also established that UD increases the expression of neurotrophins NGF and GAP-43, improves the activity of antioxidant enzymes GPx and GSH, reduces karyopyknosis within hippocampal tissue, and builds up neural-microglial density [165,166,167] within the aging brain, expanding the suggested role of UD to include the promotion of neurogenesis and neural plasticity in the hippocampus. All the while, UD was able to counteract diabetes-induced cognitive decline, rescue depressive-like behavior, and improve both spatial and associative memory [97,99,165]. Interestingly, the data suggested that the combination of UD and moderate exercise produced more prominent results, and it was able to reduce TNF-α, restore insulin levels, or increase BDNF expression in aging brains when UD or exercise alone could not [99,167].

Notably, improvements in anxiety/depressive-like behaviors have been documented after similar changes in oxidative stress markers within other brain regions, namely the prefrontal cortex and midbrain [162,166,168]. Transitioning toward the peripheral nervous system, Patel and Udayabanu (2013) determined that UD treatment could improve the hypoalgesia, or loss of pain sensation, to multiple types of stimuli, which is associated with diabetic peripheral neuropathy, noting Scopoletin as the causative agent [163,169]. With respect to neurotoxicity, UD was shown to decrease the abundance of histopathological lesions within the cerebrum and cerebellum in rabbit models, which are a defining feature of ethylene glycol poisoning [170].

As for conditions affecting motor function, UD extracts displayed antiepileptic effects due to the presence of flavonoids, which are thought to mediate convulsions through the GABAergic system [171]. Moreover, recent work by Manville et al. (2023) reported that UD components, namely gallic acid and tannic acid, can be used in the treatment of Episodic Ataxia based on their ability to ameliorate the activity of mutated Kv1.1 voltage-gated potassium channels [172]. Another condition, ischemic stroke, has also shown promising results following UD pre-treatments in mice models. In particular, XBP-1 splicing, a key process in unfolded protein response, was reduced, suggesting a prophylactic neuroprotective effect [173]. This highlights a dual role of UD in the mediation of ER stress, as other studies have linked similar treatments to the activation of apoptosis through GADD153 and increasing ER stress in cancerous cells [92].

With respect to Parkinson’s disease, recent findings demonstrated the efficacy of UD treatments in increasing the autophagic removal of α-synuclein deposits, reducing the number of Lewy bodies, and normalizing oxidative stress as a means to reduce apoptotic loss of dopaminergic neurons [168]. Concurrently, as it pertains to Alzheimer’s disease, UD root extracts were shown to improve levels of dopamine, norepinephrine, and serotonin; normalize the levels of ATP; mend memory deficits; and protect against Tau accumulation in the cortical region of the brain [166]. Combined, these results highlight the ability of UD extracts to counteract the factors contributing to neurodegenerative phenotypes. The neuroprotective activity of UD, from preliminary evidence to clinical trials, has been recently assessed by Semwal et al. [47]. 

### 7.3. Effect on the Cardiovascular System

Among the many addressed uses of *Urtica dioica* is its potential use in treating cardiovascular diseases. Earlier studies assessed its hypotensive action and reported a vasorelaxation response in vitro [174]. In parallel, using rats as test subjects, Legssyer et al. investigated the cardiovascular effects of UD and confirmed similar hypotensive results. After the injection of UD extract, the heart rate decreased, and the left ventricular pressure increased [175]. These results are contradictory to the main conclusion of their study; yet, according to the researchers, what accounts for the decrease in vascular pressure after UD injection is the bradycardia, a heart rate of fewer than 60 beats per minute [176] induced by the extract through non-adrenergic pathways, which compensate for the increase in pressure and lead to a hypotensive action that only occurs in vivo. Concurrently, data have also shown that UD extracts display anti-hypertensive properties given its angiotensin-converting enzyme (ACE) inhibition activity, which was linked to the prevalence of antioxidative compounds [13]. These findings suggest a dual role of UD in the maintenance of optimal blood pressure.

Hyperlipidemia, which refers to an excess of lipids or fat in blood, is a major cause of hepatic damage [177], and, there also, a protective effect was observed when Urtica extracts were introduced in animal models. The consumption of a UD aqueous extract by albino rats in vivo normalized the atherogenic lipoprotein phenotype and decreased total and LDL cholesterol levels specifically without having any impact on HDL cholesterol levels. The researchers concluded that UD can be used to avoid cardiovascular diseases such as hyperlipemia and any other disease associated with high LDL cholesterol levels [178].

Thrombosis and atherosclerosis are major cardiovascular diseases that are directed by platelet hyperactivity. UD has been shown to play a role in platelet aggregation; El Haouari et al. demonstrated that, after the in vitro treatment with Urtica extracts from different parts of the plants, the thrombin-induced aggregation was inhibited to various extents. This is a potential lead in using the plant to treat atherosclerosis, a main cardiovascular disease that claims a large number of victims [179]. Moreover, arteriosclerosis may be caused by oxidative stress due to the presence of free radicals. Along these lines, a recent study by Uğur and Güzel (2023) reported the efficacy of UD extract in limiting the free radical damage either by inhibiting the free radical activity or by acting as an antioxidant, hence providing major protection against arteriosclerosis [44].

### 7.4. Effect on the Reproductive System

Natural plant-derived extracts can play a significant role in hormonal regulation and overall fertility in treating conditions of the female reproductive system, including polycystic ovary syndrome (PCOS) [180]. Experimentally, dehydroepiandrosterone (DHEA)-induced PCOS mice models were used to assess the impact of UD and Lutein, a dietary carotenoid, on fertility. UD exposure was reported to decrease lipid peroxidation, increase total antioxidant capacity, normalize estrogen levels, and improve several metrics of fertility, including fertilization rates and oocyte quality. Notably, the combination of lutein and UD extract exceeded the effectiveness of each treatment individually in every observed metric except the number of oocytes and embryos [181]. In a similar study, DHEA-induced PCOS in mice was treated with a combination of UD and chamomile extract. The researchers observed that these extracts can be an effective supplement in the treatment of the symptoms of PCOS as it led to an increase in the number of normal follicles, a decrease in the number of cystic follicles, and improved overall folliculogenesis [180]. Additionally, in two other studies, UD herbal extract was able to reduce the negative impacts of retinoic acid on oocyte maturation in both in vivo and in vitro models [182,183]. The ethanolic extract of UD also exhibited pro-fertility activity, along with protective effects on ovarian cells against oxidative stress, as revealed by increased catalase activity, a major in vivo antioxidant biomarker [184]. The nettle plant is known to possess various compounds that function as aromatase inhibitors, a characteristic that has been proposed to explain its amelioration of hormonal balance. The enzyme aromatase catalyzes the conversion of androgens to estrogen [181]. This phenomenon is particularly noticeable in age-related reproductive disorders, whereby treatment with nettle powder reversed several hallmarks of sexual dysfunction in aged quails, including the estradiol/testosterone ratio, the FSH/LH ratio, egg thickness, fertility, and embryonic mortality rates [185]. Moreover, another randomized clinical trial confirmed the promising ability of UD to decrease hot flashes symptoms in menopausal women [186]. According to the European Union Herbal monograph on *Urtica dioica*, pregnant women should refrain from using UD during their pregnancy due to its ability to stimulate uterine contractions [187,188]. Moreover, it was also established that topical use of UD while breastfeeding can cause urticaria, an allergic skin rash on the mother’s skin and her breastfed infant [189]. From a different perspective, scientists also investigated the potential inhibitory effect of UD on nicotine-induced adverse implications on sperm parameters: when compared to the control group, UD significantly improved testosterone levels, seminiferous tubules diameter, and sperm cell morphology, motility, and count [190]. Taking these results together, the nettle plant is considered beneficial in ameliorating both male and female reproductive systems.

### 7.5. Effect on Digestive System

The protective effect of UD on the digestive tract was evaluated and confirmed on various gastrointestinal diseases using various models, such as mice with Dextran sulfate sodium (DSS)-induced colitis and ethanol-induced ulcer models [191,192]. This protective effect was further evaluated in a study conducted by Dakhli et al. (2023), reporting reduced DSS-induced ulcerative colitis in rat models treated with UD aqueous extract. This colo-protective effect might be attributed to its ability to reduce oxidative stress by lowering the MDA/H_2_O_2_ production, stimulating the effect of antioxidant enzymes, and reducing inflammation by decreasing CRP levels [193]. Colitis is a chronic inflammatory digestive condition of the colon’s inner lining, commonly recognized by measurement of fecal IL-1β and mucosal TNF-α. UD extract was found effective in reducing colitis clinical signs, as revealed by the significant reduction in inflammatory markers IL-1β and TNF-α in treated animals [192]. 

Addressing the intestinal flora, Fan et al. (2021) reported that UD improves gut microbiota, particularly the composition of Clostridia. Moreover, metabolic function prediction showed that UD enriches amino acid metabolism pathways that contribute to reducing body weight and increasing insulin resistance [194].

From a clinical standpoint, supplementation with a highly standardized formula containing UD, other botanical extracts, and mannitol dry extract decreased the water content in female participants with high and moderate extracellular water, while it also decreased the fat mass of those participants [195]. Furthermore, dried hydroethanolic nettle leaf extract administered to participants led to a significant reduction in high-sensitivity CRP serum levels as well as platelet count. An increase in superoxide dismutase, an enzyme responsible for free radical elimination, was also observed upon UD supplementation along with an overall health improvement in patients suffering from a coexisting inflammatory bowel disease [196].

### 7.6. Effect on the Excretory System

Several in vivo studies targeted the beneficial effect of UD on the excretory system. Al-Akash et al. (2021), using an ethylene glycol-induced (EG) Urolithiatic rabbit model, reported that the deposition of oxalate, a contributing factor in the formation of stones within the urinary tract, was reduced upon UD supplementation [149]. Additionally, the infiltration of inflammatory cells into the kidney’s interstitial tissue, as a downstream consequence of urolithiasis, was also minimized in the treated group, further suggesting the role of UD in maintaining renal function following ethylene glycol poisoning. These findings are consistent with the conclusions of KELEŞ et al. (2020), who discovered that the supplementation of UD reversed the elevation of calcium, citrate, and oxalate levels in EG mice back to normal levels. Along these lines, UD revealed potent activity in reducing the accumulation of nitrogenous contaminants such as creatinine and uric acid in blood, which are usually increased during urinary obstruction [197]. Furthermore, in the kidney tissue of EG mice, glutathione levels were lower, and malondialdehyde levels were significantly higher compared to the control and UD groups, indicating oxidative stress and highlighting the antioxidant role of UD. Moreover, after a 4-week treatment with UD, Caspase-3 activity, which leads to apoptosis in renal cells of EG mice, was almost reversed, suggesting the inhibitory role of UD against programmed cell death in kidney stone formation. UD treatment also resulted in decreased levels of inflammatory biomarkers such as TNF-α and molecules involved in inflammatory pathways, including osteopontin and collagen, which play crucial roles in disease development. The UD group exhibited a significant decrease in KIM-1, a marker for tubulointerstitial damage, with kidney tissue damage approaching control values. In contrast, the EG group showed significant tissue damage and elevated levels of KIM-1 [197].

Furthermore, recent studies investigated the synergistic effect of a multicomponent nutraceutical formulation (NF) on the formation of Calcium oxalate and uric acid crystals in the renal tubular cell line HK2. The NF consisted of UD with agents considered stone inhibitors, namely *Oreganum vulgare*, *Ceterach officinarum*, *Phyllanthus niruri*, bromelain, potassium, and magnesium citrate. In comparison to the control group, NF showed a reduction effect in CaOx crystal formation. High uric acid blood levels, known as hyperuricemia, are closely associated with stone formation via the regulatory role of URAT1 and OAT1 regulatory transporters of urate in humans, recognized as key therapeutic targets for hyperuricemia. Interestingly, exposure to UD caused a significantly downregulated mRNA expression of both transporters, further highlighting its protective effects against kidney damage [198].

### 7.7. Benign Prostatic Hyperplasia

Benign prostatic hyperplasia (BPH) is a common urological condition that affects men as they age, which can result in unpleasant urinary tract symptoms and can lead to functional urinary complications such as painful and excessive urination and recurrent urinary tract infections [199].

Alpha-blockers are currently effective treatments for BPH causing mild discomfort, resulting in a growing interest in medicinal herbs with fewer adverse effects. Indeed, UD can be used to treat urinary tract disorders related to BPH as no side effects have been reported [6,9,200,201,202]. The components of UD were described to interfere with multiple pathways involved in the development of BPH [90]. One of the most used clinical indexes in the assessment of BPH is the International Prostate Symptom Score (IPSS) [200], found to be significantly improved post-exposure to UD extract [203]. Similar results were reported by Men et al. (2016), correlating UD treatment with the improvement of quality of life among patients suffering from BPH [200,201,204]. However, there exist some inconsistencies in the literature with other studies reporting no significant amelioration of the IPSS score or clinical symptoms [200]. Karami et al. (2020) observed a weak effect of UD on the IPSS score and hypothesized that the lack of efficacy might be due to the short duration of the therapy [201]. Other studies completed the gaps by investigating other hallmarks such as the maximal urinary flow rate (Qmax), which was found to be increased along with a considerable decrease in the prostate volume upon exposure to UD [199]. Similarly, a study conducted by Sökeland (2020) confirmed a prominent increase in the Qmax of patients treated with UD. Even though there was no significant difference between the combination of UD with saw palmetto fruits and the 5-alpha reductase inhibitor, finasteride, treatment with UD was still recommended due to more favorable tolerability and safety compared with the drug [205,206]. The combination of UD with saw palmetto fruits exhibited a notable improvement in nocturnal voiding, similar to reference drugs tamsulosin and finasteride [207].

As mentioned above, the ability of UD to reduce the prostate volume might be responsible for the alleviation of BPH symptoms. In that regard, Safarinejad et al. observed a decrease in the prostatic volume when compared to the control group [200,202]. Finally, when compared to a placebo group, studies demonstrated that the postvoid residual urine (PVR) in UD-treated patients decreased, suggesting the therapeutic and beneficial activity of UD plant extract [203].

Moreover, investigations into the role of Sex Hormone-Binding Globulin (SHBG) shed further light on the mechanisms underlying the effects of UD on prostatic growth and function. Sex Hormone-Binding Globulin (SHBG) can bind to steroid hormones after adhering to prostatic stromal receptors in a ligand-free form [203]. This binding triggers an increase in intracellular cAMP production, activating protein kinase A and stimulating stromal proliferation [208]. Interestingly, lignans extracted from UD inhibited the binding of androgens to their transporter proteins SHBG, as well as the binding of these proteins to the membrane receptors of the prostate, halting their proliferative activity in prostate tissues [90,209]. Other studies further demonstrated that aqueous UD root extract effectively hindered the binding of SHBG with its receptor on male prostatic membrane surfaces [203].

As previously discussed, the antioxidant effect of UD may constitute a promising aspect of its therapeutic benefits. Indeed, collected data revealed a significant effect on oxidative and inflammatory proteins, mainly hs-CRP, MDA, and SOD, post-UD exposure [201]. The anti-inflammatory potential associated with this plant was further assessed using a combination of UD with *Serenoa repens*, which was able, through its antioxidant activity, to suppress the expression of proinflammatory pathways, including the NF-κB pathway, and to disrupt the progression of BPH. This leads to the inhibition of downstream cytokines, including IL-6 and IL-8, which have been associated with inflammation [203,210]. Congruent results were also reported for the combinations of UD with *Curcuma Longa*, *Boswellia*, *Pinus pinaster*, and soybean (*Glycine max*) [211].

## 8. Conclusions

Many researchers have explored medicinal plants for cancer treatment and prevention in recent years, particularly due to their high content in bioactive compounds, including vitamins and minerals along with a variety of secondary metabolites. Recent advances in basic research on the nettle plant have highlighted its potential use in the treatment of a wide range of diseases, including cancer. Although its potential benefits have yet to be fully defined, the existing literature has bolstered the pharmacological effects of UD both in vitro and in vivo. Several studies documented the antiproliferative, anti-inflammatory, antioxidant, analgesic, immunomodulator, and antimicrobial properties of UD along with its protective activity against hepatocellular, neurological, cardiovascular, excretory, and reproductive diseases. From the perspective of large-scale medical use, several human clinical investigations verified some of these pharmacological and nutritional benefits; however, more research is needed to confirm the perceived effects of UD and correlate its mechanism of action to its phytochemical constituents. 

## Figures and Tables

**Table 1:** The taxonomic classification of the nettle plant.

**Kingdom**	Plantae
**Division**	Magnoliophyta
**Class**	Magnoliopsida
**Order**	Rosales
**Family**	Urticaceae
**Genus**	*Urtica*
**Species**	*Urtica dioica*

**Table 2:** Overview of the in vitro apoptotic mechanisms taking place in various cancer cell lines.

Cancer Type	Cancer Cell Line	Apoptosis	Outcome	References
Prostate cancer	LNCaP	Not reported	⇩ Cellular proliferation	[52]
Prostate cancer	Prostate tissue from prostate cancer patients	Increases	⇩ ADA	[59]
Prostate cancer	PC3	Increases	⇧ Caspase 3 ⇧ Caspase 9 ⇩ Bcl-2 ⇧ G2/M arrest	[50]
Prostate cancer	LNCaP	Increases	⇧ Caspase 3 ⇧ Caspase 9 ⇧ ROS	[51]
Breast cancer	MCF-7	Increases	⇧ Calcium overload ⇧ Caspase 3 & 9 ⇧ BAX/BCL2 ⇧ DNA fragmentation	[60]
Breast cancer	MCF-7 & MDA-MB-231	Increases	⇩ MMP-1, 9, &13 ⇩ miR-21 ⇩ CXCR4 ⇧ E-cadherin ⇩ Vimentin	[66]
Breast cancer	MDA-MB-468	Increases	⇧ Pre-G0 cycle arrest ⇧ BAX/BCL2 ⇩ Snail-1 gene ⇧ G2/M cycle arrest ⇩ Cdc2 and wee1	[67]
Breast cancer	MDA-MB-231	Increases	⇧ A2B receptor activity ⇩ RAS-ERK signaling ⇩ MAPK ⇧ ODC1 overexpression ⇩P53	[62]
Blood cancer	HL-60 (AML)	Increases	⇧ G0/G1 cycle arrest ⇧ p27 ⇩ p53 ⇧ Mitochondrial membrane potential ⇧ Caspase 3, 8, & 9 ⇧ BAX/BCL2 ⇧ PTEN ⇧ BECLIN1 & LC3	[74]
Blood cancer	HL-60 (AML)	Increases	⇧ Sub-G1 cycle arrest ⇧ Caspases 3, 8, & 9 ⇩ MDM2/MDM4 ⇩ IGF1/IGF1R ⇩ PARP1 ⇩ PARP2 ⇧ PARP4	[78]
Blood cancer	U937 (AML)	Increases	⇧ Pre-G0 cycle arrest ⇧ Bax/Bcl2	[75]
Blood cancer	Jurkat (ALL)	Increases	⇧ Caspases 3, 8, & 9	[77]
Blood cancer	Raji (ALL)	Not detected	⇩ Cell proliferation	[77]
Colorectal cancer	HCT116	Increases	⇧ G2/M cycle arrest ⇧ Caspases 3 & 9 ⇩ Bcl-2	[81]
Colorectal cancer	HCT116	Increases	⇧ Bax/Bcl-2	[83]
Colorectal cancer	HCT116		⇧ Anticancer effect in combination with Wormwood extract	[89]
Bladder cancer	T24	Increases	⇧ Effect of Doxorubicin	[91]
Lung cancer	NSCLC H460, H1299, A549, and H322	Increases	⇧ GADD153 ⇧ ER stress ⇧ G2/M cycle arrest ⇧ Effect of Cisplatin	[92]

⇧: increase, ⇩: decrease

**Table 3:** Overview of the antimicrobial properties of nettle plant.

Type of Microorganism	Name of Microorganism	Effect of UD on the Microorganism	References
Pathogenic Bacteria	*Staphylococcus aureus*	Antibacterial	[69,115,121,122,123,127,129]
	*Escherichia coli*		[69,115,122,126,127,129]
	*Bacillus subtilis*		[69,115,127]
	*Pseudomonas aeruginosa*		[69,115,123,127]
	*Salmonella typhi*		[115,127]
	*Listeria monocytogenes*		[126]
	*Klebsiella pneumonia*		[115,123,125,129]
	*Pseudomonas fragi*		[124]
	*Campylobacter jejuni*		[124]
	*Shigella dysenteriae*		[115]
	*Bacillus cerus*		[123]
Non-Pathogenic Bacteria	*Lacticaseibacillus* *Strains*	No effect	[128]
	*Bifidobacterium strains*		[128]
Fungi	*Candida albicans*	Antifungal	[69,117,129]
	*Aspergillus flavus*		[69,129]
	*Candida parapsilosis*		[117]
	*Candida lipolytica*		[115]
	*Aspergillus fumigatus*		[69]
	*Aspergillus niger*		[69]
Viruses	SARS-CoV-2	Antiviral	[118,130,131]
	Rabies		[133]
Parasites	*Toxoplasma gondii*	Antiparasitic	[135]
	*Leishmania major*		[134]

## Abbreviations

AML	Acute Myeloid Leukemia
ALL	Acute Lymphocytic Leukemia
ADA	Adenosine deaminase
AKT	Protein Kinase B
BPH	Benign Prostate Hyperplasia
CNS	Central Nervous System
EG	Ethylene Glycol
FSH	Follicle Stimulating Hormone
GLUT	Glucose Transporter Type
IGF1	Insulin-like Growth Factor 1
IGF1R	Insulin-like Growth Factor 1 Receptor
IL	Interleukin
JAK2	Janus Kinase 2
LH	Luteinizing Hormone
LDL	Low-Density Lipoprotein
HDF	Human Dermal Fibroblast
HDL	High-Density Lipoprotein
HIV	Human Immunodeficiency Virus
hs-CRP	High-Sensitivity C-Reactive Protein
MMP	Matrix Metalloproteinases
NF	Nutraceutical Formulation
NFκB	Nuclear Factor Kappa-Light-Chain-Enhancer of Activated B Cells
NSCLC	Non-Small Cell Lung Carcinoma
OAT 1	Organic Anion Transporter 1
TNBC	Triple-Negative Breast Cancer
PCOS	Polycystic Ovaries Syndrome
PTEN	Phosphatase and TENsin Homolog
PI3K	Phosphoinositide 3-Kinase
ROS	Reactive Oxygen Species
SHBG	Sex Hormone-Binding Globulin
URAT1	Urate Transporter 1
VLDL	Very-Low-Density Lipoprotein
